# Generalized M-Estimation-Based Framework for Robust Guidance Information Extraction

**DOI:** 10.3390/e27121217

**Published:** 2025-11-29

**Authors:** Jiawei Ren, Xiaoyu Zhang, Shoupeng Li, Panlong Tan

**Affiliations:** 1 College of Artificial Intelligence, Nankai University, Tianjin 300350, China; renjiawei@mail.nankai.edu.cn (J.R.); tanpl@nankai.edu.cn (P.T.); 2 Institute of Modern Optics, Nankai University, Tianjin 300350, China; lishoupeng403@outlook.com

**Keywords:** guidance information estimation, generalized M-estimation, non-Gaussian noise maximum correlation entropy

## Abstract

This study tackles state estimation challenges in guidance information extraction. These challenges arise from non-Gaussian noise. We propose a robust framework to address them. The IMCIF framework effectively handles non-Gaussian noise in seeker measurements. However, noise with unstable and statistically undefined characteristics makes optimal kernel width selection difficult. This limitation compromises estimation accuracy and may even lead to filter divergence. To resolve this issue, we first linearize the nonlinear model using statistical linear regression and integrate generalized M-estimation with IMCIF. SVD is introduced to enhance numerical stability and mitigate divergence caused by suboptimal kernel width selection. Furthermore, DCS kernel function is employed to address severe non-Gaussian noise induced by large field-of-view operations and target surface reflections. A modified weight function method is proposed to preserve the L2- norm criterion while ensuring estimation accuracy under Gaussian noise. Simulations confirm the algorithm’s precision in Gaussian noise. It also maintains high accuracy under significant non-Gaussian noise, proving robustness. These improvements address both numerical stability and adaptive noise suppression, thereby enhancing system reliability across diverse interference scenarios. This work targets guidance system designers needing real-time algorithms, and filtering researchers interested in robust fusion of M-estimation and information-theoretic learning.

## 1. Introduction

The pursuit of cost-effective, high-precision modern weaponry depends on resolving critical challenges in guidance systems [1], particularly in strapdown seekers tasked with real-time estimation of line-of-sight angular rates [2]. However, during the measurement of these angular rates, two fundamental issues arise: inherent nonlinearity, where the coupling between line-of-sight and body attitude angles leads to highly nonlinear observation equations (e.g., Equation (18)), and environmental disturbances, where surface reflections on the target combined with the seeker’s wide field of view introduce wide-field interference and heavy-tailed non-Gaussian noise, posing significant challenges to accurate extraction of line-of-sight angular rates.

Kim et al. addressed coupling between line-of-sight angles and body attitude angles. Their algorithm decouples the target-seeker line-of-sight in inertial space by using coordinate transformations between body-fixed and Earth-fixed frames [3]. EKF was employed to linearize the nonlinear measurement equations [4]; however, this approach is susceptible to first-order truncation errors, which can severely degrade algorithmic accuracy. In other studies, particle filters and UKF have been applied to estimate line-of-sight angular rates, with comparative simulations under Gaussian and non-Gaussian noise conditions [5]. Results demonstrate that UKF is well-suited for strapdown seeker applications. Practical implementations show improved prediction accuracy. However, truncation errors, observational outliers, system noise uncertainties, and anomalous disturbances degrade filter performance. They cause loss of positive definiteness in the prior information matrix. To mitigate these issues, adaptive UKF variants incorporating adaptive factors have been proposed to reduce the impact of measurement errors, interference, and initial state uncertainty [6]. Nonetheless, UKF estimation accuracy deteriorates significantly under strong non-Gaussian noise. Recent studies address this limitation by replacing the Huber kernel with the Cauchy kernel for line-of-sight angular rate estimation [7]. Although low-order cost functions are computationally simple and convex, their convergence and filtering performance are markedly reduced in scenarios with intense non-Gaussian noise.

Non-Gaussian noise severely impacts Gaussian filter efficacy. To enhance robustness against such noise, correlation-robust filters have been developed. Current robust filtering approaches focus on three strategies: The first is to revise the updating process of standard Kalman filter based on GML to obtain the desired robustness [8,9,10,11,12,13,14]. The second method is to replace the original Gaussian probability distribution with the Student-t distribution with thick tail; that is, the Student-t distribution is used to model the non-Gaussian distribution, so as to obtain better filtering accuracy by better approximating the actual distribution [15,16,17,18,19,20]. The third approach incorporates ITL to reformulate the objective function using MCC and MEE, enhancing the robustness of Kalman filtering against large outliers through these information-theoretic optimization criteria [21,22,23,24]. Strapdown seekers are particularly vulnerable to strong non-Gaussian noise. In this paper, an iterative maximum correlation entropy square root information filter based on a generalized M estimation is proposed to reduce seeker interference from external disturbances. An enhanced weight function modification method is introduced, preserving the L2- norm criterion while ensuring estimation accuracy under Gaussian noise. Simulations demonstrate the algorithm’s precision under Gaussian conditions. The main contributions are as follows:

(1) Development of IMMCSRIF using generalized M-estimation.
(2) Design of an Adaptive Weight Function optimizing L2- norm criterion performance under Gaussian noise.
(3) Verify the theoretical prediction through simulation.

The remaining sections of this paper are organized as follows. Section 2 offers a problem formulation. Section 3 offers a concise introduction to the fundamental concepts of Gaussian kernel maximum correlation entropy and DCS. Section 4 provides a brief review of the line-of-sight angular rate decoupling algorithm. Section 5 presents the IMMCSRIF framework and an improved modified weight function. Section 6 presents simulation comparisons of various filtering methods using Monte Carlo methods. Finally, Section 7 presents the conclusions of this study.

## 2. Problem Formulation

Consider the strapdown seeker guidance system with the state-space model:

(1) $$\left\{\begin{array}{c} x_{k}=f\left(x_{k-1}\right)+\omega_{k-1}\\ z_{k}=g\left(x_{k}\right)+\nu_{k} \end{array}\right.,$$

where $x_{k\;}\in R^{n}$ is the state vector (LOS angles and angles rates), $z_{k\;}\in R^{m}$ is the measurement. The process noise $\omega_{k-1}\sim N\left(0,Q\right)$ is Gaussian noise, while the measurement noise $\nu_{k}$ follows a heavy-tailed distribution:

$$\nu_{k}\sim \left(1-\varepsilon \right)N\left(0,\sigma_{1}^{2}\right)+\varepsilon N\left(0,\sigma_{2}^{2}\right),\sigma_{2}\gg \sigma_{1}$$

The core challenge is to design an estimator ${\hat{x}}_{k}=F\left(z_{1:k}\right)$ that minimizes $E\left[{\left\|x_{k}-{\hat{x}}_{k}\right\|}^{2}\right]$ under:

(1) Strong nonlinearity: $g\left(\cdot \right)$ is non-differentiable (e.g., Equation (18)).
(2) Non-Gaussian noise: ε>0 causes biased covariance estimation.
(3) Numerical instability: $P_{k|k-1}$ under model mismatch.

## 3. Preliminaries

### 3.1. Maximum Correlation Entropy Cost Function

For random variables *X* and *Y*, correntropy defines their similarity using the following equation:

(2) $$V\left(X,Y\right)=E\left(\kappa \left(X,Y\right)\right)=\int_{-\infty }^{+\infty }\int_{-\infty }^{+\infty }\kappa \left(x,y\right)p_{X,Y}\left(x,y\right)dxdy,$$

where E(⋅) denotes the expectation operator, κ(⋅) denotes the kernel function, the support of *x* and *y* is $\left\{x\in R,y\in R\right\}$, and $p_{X,Y}(x,y)$ denotes the joint density function of *X* and *Y*, where κ(⋅) needs to satisfy three properties [25,26,27,28,29,30]:

(1) Positive definiteness: the kernel matrix must be semi-positive definite.
(2) Symmetry: $\kappa \left(x,y\right)=\kappa \left(y,x\right)$.
(3) Decaying Influence: $\kappa \left(x,y\right)$ decreases as the error $\left|x-y\right|$ increases, suppressing the effect of outliers.

The Gaussian kernel works as the kernel function and is given by the following equation [21]:

(3) $$\kappa (x,y)=G_{\sigma }(e)=\exp(-\frac{e^{2}}{2\sigma^{2}}),$$

where e=x−y represents the error between X and Y, and σ>0 is the kernel bandwidth. In practical applications, since the true joint distribution $p_{X,Y}$ is often unavailable [31,32], e is typically approximated as the difference between measurements and their estimates.

In practice, sample estimates are often used. Equation (3) can therefore be estimated as a Monte Carlo type:

(4) $$\hat{V}\left(X,Y\right)=\frac{1}{N}\sum_{k=1}^{N}\kappa \left(x_{k},y_{k}\right).$$

With $\{x_{k},{y_{k}\}}_{k=1}^{N}$ being N samples drawn from $p_{X,Y}$. $\hat{V}(X,Y)$ is a Monte Carlo type estimator.

### 3.2. DCS

In the field of robot vision SLAM, a cost function has been proposed that satisfies the robust DCS kernel requirement by adjusting a single parameter, as follows [33,34]:

(5) $$\rho_{S}(\xi_{k,i})=s_{i}^{2}\xi_{k,i}^{2}+\theta {\left(1-s_{i}\right)}^{2}.$$

where $s_{i}$ denotes the switchable variable and θ denotes the tuning parameter of the DCS kernel. $\xi_{k,i}$ is the residual difference between the observed and predicted quantities.

When the estimate converges, the partial derivative of the cost function with respect to the switchable variable should be zero. Therefore, the optimal closed-form solution for $s_{i}$ is given by

(6) $$\frac{\partial \rho_{S}(\xi_{k,i})}{\partial s_{i}}=2s_{i}\xi_{k,i}^{2}-2\theta (1-s_{i})=0,$$

(7) $$s_{i}=\frac{\theta }{\theta +\xi_{k,i}^{2}}.$$

By substituting Equation (7) into Equation (3), we derive the SC cost function with a single tuning parameter as follows:

(8) $$\rho_{S}(\xi_{k,i})={\left(\frac{\theta }{\theta +\xi_{k,i}^{2}}\right)}^{2}\xi_{k,i}^{2}+\theta {\left(1-\frac{\theta }{\theta +\xi_{k,i}^{2}}\right)}^{2}=\frac{\theta \xi_{k,i}^{2}}{\theta +\xi_{k,i}^{2}}.$$

Based on Equation (8), the limit value of the SC cost function can be obtained as follows:

(9) $$\underset{\xi_{k,i}\to \pm \infty }{lim}\rho_{S}(\xi_{k,i})=\underset{\xi_{k,i}\to \pm \infty }{lim}\frac{\theta }{\theta /\xi_{k,i}+1}=\theta .$$

As can be observed from Equation (9), an upper bound for $\rho_{S}$ is θ; that is, $\rho_{S}\leq \theta$. Therefore, the minimum of s the switchable variable is

(10) $$s=min\left(1,\frac{2\theta }{\theta +\xi_{k,i}^{2}}\right).$$

Based on the literature derivation [35], DCS can be applied to the iterative reweighted least squares algorithm and generalized M estimation to make the estimator robust to outliers. Therefore, Equation (10) can be used as a robust kernel function. The DCS weighting function $\psi_{D}$ has the following form:

(11) $$\psi_{D}\left(\xi_{k,i}\right)=s_{i}^{2}=min\left(1,\frac{4\theta }{{\left(\theta +\xi_{k,i}^{2}\right)}^{2}}\right)=\left\{\begin{array}{c} \;1,\;for\;\xi_{k,i}^{2}<\theta \\ \frac{4\theta }{{\left(\theta +\xi_{k,i}^{2}\right)}^{2}},\;for\xi_{k,i}^{2}\geq \theta \end{array}\right..\;$$

**Proposition** **1**  **(DCS** **Optimality). ***The DCS weight $\psi_{D}(\xi )$ in Equation (11) is the minimizer of the majorized surrogate function:*

(12) $$\psi_{D}(\xi )=arg\underset{s}{min}\left[s^{2}\xi^{2}+\theta (1-s{)}^{2}\right].$$

**Proof.**  Setting the derivative of the surrogate with regard to s to zero:

(13) $$\frac{\partial }{\partial s}\left(s^{2}\xi^{2}+\theta (1-s{)}^{2}\right)=2s\xi^{2}-2\theta (1-s)=0,$$

yields $s=\frac{\theta }{\theta +\zeta^{2}}$. Substituting into Equation (8) gives Equation (11). This ensures $\psi_{D}(\xi )$ preserves the L2- norm for small ξ while saturating for large ξ. □

## 4. Line-of-Sight Angle Rate Decoupling Equation

First, we review the line-of-sight angle rate decoupling algorithm, which determines the line-of-sight angle rate using strapdown seeker measurements. Because the strapdown imaging seeker can directly detect the azimuth and elevation angles in the body-frame coordinate system, we may utilize these data as observable quantities. The connection between these measurements and the state variables must be determined. In both the line-of-sight and body-frame coordinate systems, we can represent the location of the target as ${\left[r,0,0\right]}^{T}$. The coordinates of the target in both the inertial coordinate system and the body-frame coordinate system are as follows [5]:

(14) $$\left[\begin{array}{c} x_{b}\\ y_{b}\\ z_{b} \end{array}\right]=C_{l}^{b}\left[\begin{array}{c} r\\ 0\\ 0 \end{array}\right]=\left[\begin{array}{c} \mathrm{rcos}\left(q_{\alpha }\right)\cos\left(q_{\beta }\right)\\ \mathrm{rsin}\left(q_{\alpha }\right)\\ -\mathrm{rcos}\left(q_{\alpha }\right)\sin\left(q_{\beta }\right) \end{array}\right],$$

(15) $$\left[\begin{array}{c} x_{f}\\ y_{f}\\ z_{f} \end{array}\right]=C_{s}^{f}\left[\begin{array}{c} r\\ 0\\ 0 \end{array}\right]=\left[\begin{array}{c} \mathrm{rcos}\left(q_{\gamma }\right)\cos\left(q_{\lambda }\right)\\ \mathrm{rsin}\left(q_{\gamma }\right)\\ -\mathrm{rcos}\left(q_{\gamma }\right)\sin\left(q_{\gamma }\right) \end{array}\right].$$

If we multiply both sides of the equation by matrix $C_{f}^{b}$, we have

(16) $$\left[\begin{array}{c} x_{b}\\ y_{b}\\ z_{b} \end{array}\right]=C_{f}^{b}\left[\begin{array}{c} x_{f}\\ y_{f}\\ z_{f} \end{array}\right]=C_{f}^{b}C_{s}^{f}\left[\begin{array}{c} r\\ 0\\ 0 \end{array}\right]=C_{f}^{b}\left[\begin{array}{c} \mathrm{rcos}\left(q_{\gamma }\right)\cos\left(q_{\lambda }\right)\\ \mathrm{rsin}\left(q_{\gamma }\right)\\ -\mathrm{rcos}\left(q_{\gamma }\right)\sin\left(q_{\gamma }\right) \end{array}\right],$$

If we consider Equations (14) and (16) simultaneously, we obtain

(17) $$C_{f}^{b}\left[\begin{array}{c} \mathrm{rcos}\left(q_{\gamma }\right)\cos\left(q_{\lambda }\right)\\ \mathrm{rsin}\left(q_{\gamma }\right)\\ -\mathrm{rcos}\left(q_{\gamma }\right)\sin\left(q_{\gamma }\right) \end{array}\right]=\left[\begin{array}{c} \mathrm{rcos}\left(q_{\alpha }\right)\cos\left(q_{\beta }\right)\\ \mathrm{rsin}\left(q_{\alpha }\right)\\ -\mathrm{rcos}\left(q_{\alpha }\right)\sin\left(q_{\beta }\right) \end{array}\right].$$

From Equation (14), we have

(18) $$\left\{\begin{array}{c} q_{\alpha }=\arcsin(C_{21}cq_{\gamma }cq_{\lambda }+C_{22}sq_{\gamma }-C_{23}cq_{\gamma }sq_{\lambda })\\ q_{\beta }=-\arctan(\frac{C_{31}cq_{\lambda }+C_{32}tq_{\gamma }-C_{33}sq_{\lambda }}{C_{11}cq_{\lambda }+C_{12}tq_{\gamma }-C_{13}sq_{\lambda }}) \end{array}\right..$$

In this equation, $C_{ij}$ denotes the element in the i-th row and j-th column of the transformation matrix $C_{f}^{b}$, which transforms coordinates from the inertial coordinate system $O_{f}-x_{f}y_{f}z_{f}$ to the body-frame coordinate system $O_{b}-x_{b}y_{b}z_{b}$. Additionally, s, c, and t denote the trigonometric functions sin, cos, and tan, respectively.

**Figure 1:**
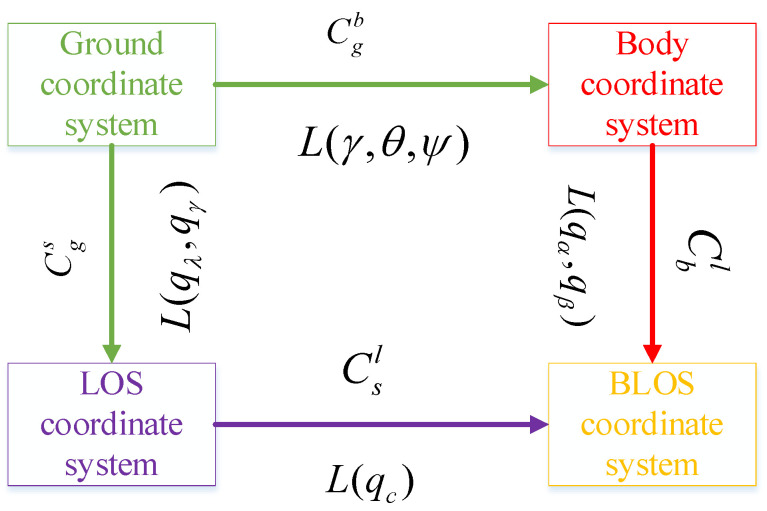
Rotation relationships among coordinate systems. In Figure 1, LOS is represented as the line-of-sight coordinate system, and BLOS is represented as the body line of sight coordinate system. $L\left(\cdot \right)$ is represented by the coordinate system transformation matrix.

The rotational relationships among the frames are shown in Figure 1. The coordinate transformation matrix in the figure is as follows:

$$C_{g}^{b}=\left[\begin{array}{ccc} c\theta c\psi & s\theta & -c\theta s\psi \\ s\phi s\psi & c\phi c\theta & s\phi c\theta +c\phi s\theta s\psi \\ c\phi s\psi +s\phi s\theta c\psi & -s\phi c\theta & c\phi c\psi -s\phi s\theta s\psi \end{array}\right];\;C_{s}^{l}=\left[\begin{array}{ccc} 1 & 0 & 0\\ 0 & cq_{c} & sq_{c}\\ 0 & -sq_{c} & cq_{c} \end{array}\right];\;C_{g}^{s}=\left[\begin{array}{c} cq_{\gamma }cq_{\lambda }\;sq_{\gamma }\;-cq_{\gamma }sq_{\lambda }\\ -sq_{\gamma }cq_{\lambda }\;cq_{\gamma }\;sq_{\gamma }sq_{\lambda }\\ sq_{\lambda }\;0\;cq_{\lambda } \end{array}\right];C_{b}^{l}=\left[\begin{array}{ccc} cq_{\alpha }cq_{\beta } & sq_{\alpha } & -cq_{\alpha }sq_{\beta }\\ -sq_{\alpha }cq_{\beta } & cq_{\alpha } & sq_{\alpha }sq_{\beta }\\ sq_{\beta } & 0 & cq_{\beta } \end{array}\right].$$

where θ, ϕ, and ψ denote pitch, roll, and yaw angles, respectively. $q_{\alpha }$ denotes the elevation angle of the line of sight, and $q_{\beta }$ denotes the azimuth angle of the line of sight. $q_{\gamma }$ denotes the elevation angle of the line of sight, while $q_{\lambda }$ denotes the azimuth angle of the line of sight. $q_{c}$ denotes the angle of line-of-sight transformation. Furthermore, s and c denote the sine and cosine functions in trigonometry, respectively.

Based on the literature [6], let $x=[q_{\gamma }\;{\dot{q}}_{\gamma }q_{\lambda }{\dot{q}}_{\lambda }]$, and we can establish the state equation as follows:

(19) $$\left\{\begin{array}{c} {\dot{x}}_{1}=x_{2}\\ {\dot{x}}_{2}=-\frac{2\dot{r}}{r}x_{2}-x_{4}^{2}\sin\left(x_{1}\right)\cos\left(x_{1}\right)+\frac{a_{y_{s}}}{r}\\ {\dot{x}}_{3}=x_{4}\\ {\dot{x}}_{4}=2x_{2}x_{4}tan\left(x_{1}\right)-\frac{2\dot{r}}{r}x_{4}-\frac{a_{z_{s}}}{rcos\left(x_{1}\right)} \end{array}\right.,$$

where $x_{i}$ represents the i-th element of the state variable vector for i = 1, 2, 3, 4. $a_{y_{s}}$ represents the Y-axis relative acceleration component of the missile and the target in the line-of-sight coordinate system. $a_{z_{s}}$ represents the Z-axis relative acceleration component of the missile and the target in the line-of-sight coordinate system. It is worth noting that $q_{\gamma }$ equals $\frac{\pi }{2}$ should be avoided when designing the missile trajectory. This is due to the appearance of $tan\left(q_{\gamma }\right)$ and $cos\left(q_{\gamma }\right)$ in the formula, which may cause infinite value when solving the line-of-sight angle rate, so that the equation cannot be used.

## 5. Improved IMMCSRIF

### 5.1. Development of IMMCSRIF

Consider the following nonlinear discrete system:

(20) $$\left\{\begin{array}{c} x_{k}=f\left(x_{k-1}\right)+\omega_{k-1}\\ z_{k}=g\left(x_{k}\right)+\nu_{k} \end{array}\right.,$$

where $f\left(\cdot \right)$ and $g\left(\cdot \right)$ denote the structural parameters of the nonlinear systems, and $x_{k}$ and $z_{k}$ denote the n-dimensional state vector and m-dimensional measurement vector, respectively. $\omega_{k-1}$ and $\nu_{k}$ denote the l-dimensional noise vector of the system and the m-dimensional measurement noise vector, respectively. Furthermore, the following relationships hold:

$$E[\omega_{k}]=0,E[\nu_{k}]=0,E[\omega_{k}\omega_{k}^{T}]=Q_{k}\delta_{kj},E[\nu_{k}\nu_{k}^{T}]=R_{k}\delta_{kj},E[\omega_{k}\nu_{k}^{T}]=0,$$

where $Q_{k}$ and $R_{k}$ denote the system noise covariance matrix and observation noise covariance matrix, respectively.

First, the one-step predicted value and square root factor of the covariance matrix of the prediction error at time k are calculated:

(1) The one-step predicted value ${\hat{x}}_{k|k-1}$ is calculated as follows:
  (21) $$\left\{\begin{array}{c} {\hat{X}}_{i,k|k-1}=f\left(X_{i,k-1}\right)\\ \;{\hat{x}}_{k|k-1}=\sum_{i=1}^{2n}w_{i}{\hat{X}}_{i,k|k-1} \end{array}\right.,$$
  where $w_{i}$ is expressed as the corresponding weight of point set, the expression of which is shown in Algorithm 1.
(2) The square root factor of the covariance matrix of the prediction error $S_{k|k-1}$ is calculated as follows:
  (22) $$\left\{\begin{array}{c} \left[{\tilde{\chi }}_{k|k-1},S_{Q_{k}}\right]\overset{SVD}{\to }U_{P_{k|k-1}}D_{P_{k|k-1}}V_{P_{k|k-1}}^{T}\\ \;S_{k|k-1}\overset{SVD}{\to }U_{P_{k|k-1}}\sqrt{D_{P_{k|k-1}}} \end{array}\right.,$$
  (23) $$S_{y_{k|k-1}}=S_{k|k-1}^{-T},$$
  where ${\tilde{\chi }}_{k|k-1}$ denotes the difference between the volume point of the state variable and the predicted value of the estimated state, while $S_{Q_{k}}$ is obtained by performing SVD on the system noise covariance matrices $Q_{k}$.

Note: The SVD decomposition ensures numerical stability by avoiding ill-conditioned covariance matrices. Specifically:

The prior covariance square root is updated as $S_{k|k-1}=U_{P}\sqrt{D_{P}}$, guaranteeing positive semi-definiteness.

The condition number of $S_{k|k-1}$ is bounded by $\kappa \left(S_{k|k-1}\right)\leq \sqrt{\kappa \left(D_{P}\right)},$ preventing divergence under suboptimal kernel widths.

The nonlinear measurement equation can be linearized by applying statistical linear regression [36]. Hence, the statistical linear regression of the observation model can be expressed as follows:

(24) $$z_{k}=G_{a,k}x_{k}+G_{b,k}+v_{k}^{*},$$

where

(25) $$\left\{\begin{array}{c} G_{a,k}={\tilde{\chi }}_{k|k-1}Z_{k|k-1}^{T}Y_{k|k-1}\\ G_{b,k}={\hat{z}}_{k|k-1}-G_{a,k}{\hat{x}}_{k|k-1} \end{array}\right.,$$

where $Z_{k|k-1}$ denotes the difference between the volume point of the measured quantity and the predicted value. The noise covariance corresponding to the linearized measurement model is given by

(26) $$S_{R_{k|k-1}^{*}}=Z_{k|k-1}-G_{a,k}S_{k|k-1}+S_{R_{k}},$$

where $S_{R_{k}}$ is obtained by performing an SVD on the system noise covariance matrices $R_{k}$.

The aforementioned method employs linear statistical regression to convert a nonlinear model into a linear one [37], thereby facilitating the utilization of the GML estimator [11]. The modified steps are as follows:

(27) $$\gamma_{k}\left[\begin{array}{c} {\hat{x}}_{k|k-1}\\ z_{k}-G_{b,k} \end{array}\right]=\gamma_{k}\left[\begin{array}{c} E\\ G_{a,k} \end{array}\right]x_{k}+\gamma_{k}\left[\begin{array}{c} -\delta_{k}\\ v_{k}^{*} \end{array}\right],$$

where $\delta_{k}$ denotes the prior residuals, namely $\delta_{k}=x_{k}-{\hat{x}}_{k\left|k-1\right.}$, and the covariance of the residuals is denoted by ${\left[-\delta_{k}v_{k}^{*}\right]}^{T}$. The matrix $\gamma_{k}$ is obtained from ${\;\gamma }_{k}=\left[\begin{array}{cc} S_{y_{k|k-1}} & 0\\ 0 & S_{R_{k|k-1}^{*}}^{-1} \end{array}\right]$.

Equation (27) can be expressed in a compact form as follows:

(28) $$\beta_{k}=M_{k}x_{k}+{\tilde{\xi }}_{k}.$$

In this case, we have $E[{\tilde{\xi }}_{k}{\tilde{\xi }}_{k}^{T}]=1$, and Equation (28) constitutes the standardized nonlinear regression model. Let ${\tilde{\xi }}_{k}=\beta_{k}-M_{k}x_{k}$ be the standardized residuals, where $M_{k}$ is the normalized observation matrix, ${\tilde{\xi }}_{k}$ is the normalized residual, and $\beta_{k}$ is the normalized observation vector.

In contrast to previous studies [7], this study introduces the maximum correlation entropy cost function. When handling non-Gaussian environmental noise, this function can better suppress the significant outliers introduced by non-Gaussian noise, thereby minimizing their impact on filter weights and enhancing the overall robustness of the filtering algorithm.

For a strong nonlinear system, it is generally believed that the posterior estimation is better than the prior estimation [38]. Therefore, to build a more accurate linear regression model, it is more reasonable to use the posterior estimation results to constantly update the matrix $M_{k}$. Take the *j* iteration loop, for example. In the IMCKF, the cost function is as follows [31]:

(29) $$J_{L}^{\left(j\right)}(x_{k})=\frac{1}{L}\sum_{i=1}^{L}G_{\sigma }\left(\beta_{i,k}-M_{i,k}^{\left(j\right)}x_{k}\right),$$

where $\beta_{i,k}$ denotes the i-th element of $\beta_{k}$, and $M_{i,k}$ denotes the i-th row of $M_{k}$. $G_{\sigma }\left(\cdot \right)$ denotes the Gaussian kernel function described in Equation (5). L=n+m, where n and m denote the dimensions of the state vector and the observation vector, respectively.

In the IMMCSRIF, the weighting factors are added to the equation of the cost function under the IMCKF. The cost function of the robust update is as follows:

(30) $$J_{L}^{\left(j\right)}(x_{k})=\frac{1}{L}\sum_{i=1}^{L}\omega_{i,k}^{\left(j\right)}G_{\sigma }\left(\beta_{i,k}-M_{i,k}^{\left(j\right)}x_{k}\right).$$

**Theorem** **1.** *The state estimate* $x_{k}$ *minimizing the cost function (27) satisfies the first-order optimality condition:*

(31) $$\frac{\partial J_{L}^{\left(j\right)}(x_{k})}{\partial x_{k}}=-\sum_{i=1}^{L}\omega_{i,k}^{\left(j\right)}G_{\sigma }\left({\tilde{\xi }}_{i,k}\right)M_{i,k}^{(j)T}\;=\;0.$$

*where *${\tilde{\xi }}_{i,k}=\beta_{i,k}-M_{i,k}^{\left(j\right)}x_{k}$*. Rearranging terms yields*

(32) $$\sum_{i=1}^{L}\omega_{i,k}^{\left(j\right)}G_{\sigma }\left({\tilde{\xi }}_{i,k}\right)M_{i,k}^{(j)T}M_{i,k}^{\left(j\right)}x_{k}=\sum_{i=1}^{L}\omega_{i,k}^{\left(j\right)}G_{\sigma }\left({\tilde{\xi }}_{i,k}\right)M_{i,k}^{(j)T}\beta_{i,k}.$$

*This is equivalent to the closed-form solution (34)–(36).*

Theoretical Motivation. The proposed IMMCSRIF framework minimizes a robustified correntropy objective:

(33) $$\underset{x_{k}}{min}\sum_{i=1}^{L}\omega_{i,k}^{\left(j\right)}\rho \left(\xi_{i,k}\right),\rho \left(\xi \right)=exp\left(-\frac{\xi^{2}}{2\sigma^{2}}\right),$$

where ρ(ξ) is the correntropy-induced loss function, and $\omega_{i,k}^{\left(j\right)}$ are weights from generalized M-estimation. This dual mechanism suppresses outliers via $\omega_{i,k}$ while preserving higher-order moments via ρ(ξ).

Then, we can calculate the optimal estimate of $x_{k}$:

(34) $$\frac{\partial J_{L}^{\left(j\right)}(x_{k})}{\partial x_{k}}=\sum_{i=1}^{L}[\omega_{i,k}^{\left(j\right)}G_{\sigma }\left(\beta_{i,k}-M_{i,k}^{\left(j\right)}x_{k}\right){\left(M_{i,k}^{\left(j\right)}\right)}^{T}\left(\beta_{i,k}-M_{i,k}^{\left(j\right)}x_{k}\right)]=0,$$

(35) $$x_{k}={\left(\sum_{i=1}^{L}[\omega_{i,k}^{\left(j\right)}G_{\sigma }\left(\beta_{i,k}-M_{i,k}^{\left(j\right)}x_{k}\right){\left(M_{i,k}^{\left(j\right)}\right)}^{T}M_{i,k}^{\left(j\right)}\right)}^{-1}\times \left(\sum_{i=1}^{L}[\omega_{i,k}^{\left(j\right)}G_{\sigma }\left(\beta_{i,k}-M_{i,k}^{\left(j\right)}x_{k}\right)\beta_{i,k}\right),$$

(36) $$x_{k}={\left({\left(M_{k}^{\left(j\right)}\right)}^{T}\omega_{k}^{\left(j\right)}\Lambda_{k}^{\left(j\right)}M_{k}^{\left(j\right)}\right)}^{-1}{\left(M_{k}^{\left(j\right)}\right)}^{T}\omega_{k}^{\left(j\right)}\Lambda_{k}^{\left(j\right)}\beta_{k}={\left({\left(M_{k}^{\left(j\right)}\right)}^{T}{\bar{\Lambda }}_{k}^{\left(j\right)}M_{k}^{\left(j\right)}\right)}^{-1}{\left(M_{k}^{\left(j\right)}\right)}^{T}{\bar{\Lambda }}_{k}^{\left(j\right)}\beta_{k},$$

where $\Lambda_{k}^{\left(j\right)}=\left[\begin{array}{cc} \Lambda_{x,k}^{\left(j\right)} & 0\\ 0 & \Lambda_{y,k}^{\left(j\right)} \end{array}\right]$, $\Lambda_{x,k}^{\left(j\right)}=diag(G_{\sigma }({\tilde{\xi }}_{1,k}^{\left(j\right)}),\cdots ,G_{\sigma }({\tilde{\xi }}_{n,k}^{\left(j\right)}()$, $\Lambda_{y,k}^{\left(j\right)}=diag(G_{\sigma }({\tilde{\xi }}_{n+1,k}^{\left(j\right)}),\cdots ,G_{\sigma }({\tilde{\xi }}_{n+m,k}^{\left(j\right)}))$,

(37) $${\bar{\Lambda }}_{x,k}^{\left(j\right)}=\omega_{x,k}^{\left(j\right)}\Lambda_{x,k}^{\left(j\right)}=\left(diag\left(\omega_{1,k}^{\left(j\right)},\cdots ,\omega_{n,k}^{\left(j\right)}\right)\right)\times \left(diag\left(G_{\sigma }\left({\tilde{\xi }}_{1,k}^{\left(j\right)}\right),\cdots ,G_{\sigma }\left({\tilde{\xi }}_{n,k}^{\left(j\right)}\right)\right)\right),$$

(38) $${\bar{\Lambda }}_{y,k}^{\left(j\right)}=\omega_{y,k}^{\left(j\right)}\Lambda_{y,k}^{\left(j\right)}=\left(diag\left(\omega_{n+1,k}^{\left(j\right)},\cdots ,\omega_{n+m,k}^{\left(j\right)}\right)\right)\times \left(diag\left(G_{\sigma }\left({\tilde{\xi }}_{n+1,k}^{\left(j\right)}\right),\cdots ,G_{\sigma }\left({\tilde{\xi }}_{n+m,k}^{\left(j\right)}\right)\right)\right),$$

(39) $$\left\{\begin{array}{c} S_{m,y_{k|k-1}}^{\left(j\right)}={\left({\bar{\Lambda }}_{x,k}^{\left(j\right)}\right)}^{\frac{1}{2}}S_{y_{k|k-1}}^{\left(j\right)}\\ S_{m,R_{k|k-1}^{*}}^{\left(j\right)}={\left({\bar{\Lambda }}_{y,k}^{\left(j\right)}\right)}^{\frac{1}{2}}S_{R_{k|k-1}^{*}}^{\left(j\right)} \end{array}\right.,$$

where ${\bar{\Lambda }}_{x,k}^{\left(j\right)}$ and ${\bar{\Lambda }}_{y,k}^{\left(j\right)}$ denote the state vector and observation vector components of ${\bar{\Lambda }}^{\left(j\right)}$, respectively. Then, we obtain the predictive information vector ${\hat{y}}_{k\left|k-1\right.}^{\left(j\right)}$ and the square root factor of the covariance matrix $S_{k}^{\left(j\right)}$:

(1) The predictive information vector ${\hat{y}}_{k\left|k-1\right.}^{\left(j\right)}$ is calculated as follows:
  (40) $${\hat{y}}_{k\left|k-1\right.}^{\left(j\right)}=S_{m,y_{k|k-1}}^{\left(j\right)}{\left(S_{m,y_{k|k-1}}^{\left(j\right)}\right)}^{T}{\hat{x}}_{k|k-1}.$$
(2) The square root factor of the covariance matrix of the prediction error $S_{k}^{\left(j\right)}$ is calculated as follows:
  (41) $$\left\{\begin{array}{c} \left[S_{m,y_{k|k-1}}^{\left(j\right)},G_{a,k}^{\left(j\right)},S_{m,R_{k|k-1}^{*}}^{\left(j\right)}\right]\overset{SVD}{\to }U_{Y_{k}^{\left(j\right)}}D_{Y_{k}^{\left(j\right)}}{\left(V_{Y_{k}^{\left(j\right)}}\right)}^{T}\\ \;S_{y_{k}}^{\left(j\right)}\overset{SVD}{\to }U_{Y_{k}^{\left(j\right)}}\sqrt{D_{Y_{k}^{\left(j\right)}}} \end{array}\right.,$$
  (42) $$S_{k}^{\left(j\right)}=S_{y_{k}}^{(j)-T},$$

After the square root factor of the information matrix is obtained, the information contribution vector $i_{k}^{\left(j\right)}$ can be calculated as follows:

(43) $$i_{k}^{\left(j\right)}={\left(G_{a,k}^{\left(j\right)}\right)}^{T}S_{m,R_{k|k-1}^{*}}^{\left(j\right)}{\left(S_{m,R_{k|k-1}^{*}}^{\left(j\right)}\right)}^{T}\left(z_{k}-G_{b,k}\right).$$

The corresponding information vector update value ${\hat{y}}_{k}^{\left(j+1\right)}$ is

(44) $${\hat{y}}_{k}^{\left(j+1\right)}={\hat{y}}_{k\left|k-1\right.}^{\left(j\right)}+i_{k}^{\left(j\right)}.$$

The IMMCSRIF is summarized and the algorithm flow table is shown in Algorithm 1.
**Algorithm 1:** Summary of the IMMCSRIF algorithm1. Determine the initial filtering parameters$\;{\hat{x}}_{0}=E[x_{0}]$, $\;P_{0}=E[(x_{0}-{\hat{x}}_{0}){\left(x_{0}-{\hat{x}}_{0}\right)}^{T}]$. $\;Y_{0}=P_{0}^{-1}$, 2. Prediction(1)Calculate the volume point at time *k* − 1:$\begin{array}{c} \;Y_{k-1}\overset{SVD}{\to }U_{Y_{k-1}}D_{Y_{k-1}}^{2}V_{Y_{k|k-1}}^{T},\\ \;Q_{k-1}\overset{SVD}{\to }U_{Q_{k-1}}\left[\begin{array}{cc} S_{Q_{k-1}} & 0\\ 0 & 0 \end{array}\right]V_{Q_{k-1}}^{T},\\ \;S_{Q_{k-1}}\overset{SVD}{\to }U_{Q_{k-1}}\left[\begin{array}{cc} \sqrt{S_{Q_{k-1}}} & 0\\ 0 & 0 \end{array}\right],\\ \;S_{k-1}=S_{y_{k-1}}^{-T},\\ \;X_{i,k-1}={\hat{x}}_{k-1}+U_{Y_{k-1}}D_{Y_{k-1}}^{-1}\xi_{i}, \end{array}$ $\mathrm{where}\;S_{k-1}$$\;\mathrm{is}\;\mathrm{obtained}\;\mathrm{by}\;\mathrm{the}\;\mathrm{Cholesky}\;\mathrm{decomposition}\;\mathrm{of}\;P_{k-1}$$;\;\mathrm{point}\;\mathrm{set}\;\xi_{i}\;$$\mathrm{and}\;\mathrm{its}\;\mathrm{corresponding}\;\mathrm{weight}\;w_{i}$ are
$\begin{array}{c} \;\xi_{i}=\sqrt{n}{\left[1\right]}_{i},\\ \;w_{i}=\frac{1}{2n}. \end{array}$

(2)Calculate the one-step predicted value and its covariance at time k:
$\begin{array}{c} \;{\hat{X}}_{i,k\left|k-1\right.}=f\left(X_{i,k-1}\right),\\ \;{\hat{x}}_{k|k-1}=\sum_{i=1}^{2n}w_{i}{\hat{X}}_{i,k|k-1}. \end{array}$

(3)Calculate the covariance matrix square root factor of the prediction error: 
$\begin{array}{c} \;{\tilde{\chi }}_{k|k-1}=\sqrt{w_{i}}\left[{\hat{X}}_{1,k|k-1}-{\hat{x}}_{k|k-1},{\hat{X}}_{2,k|k-1}-{\hat{x}}_{k|k-1},\cdots ,{\hat{X}}_{2n,k|k-1}-{\hat{x}}_{k|k-1}\right],\\ \;\left[{\tilde{\chi }}_{k\left|k-1\right.},S_{Q_{k-1}}\right]\overset{SVD}{\to }U_{P_{k\left|k-1\right.}}\left[\begin{array}{cc} S_{P_{k\left|k-1\right.}} & 0\\ 0 & 0 \end{array}\right]V_{P_{k\left|k-1\right.}}^{T},\\ \;S_{k|k-1}\overset{SVD}{\to }U_{P_{k\left|k-1\right.}}\left[\begin{array}{cc} \sqrt{S_{P_{k\left|k-1\right.}}} & 0\\ 0 & 0 \end{array}\right],\\ \;S_{y_{k|k-1}}=S_{k|k-1}^{-T},\\ \;{\hat{y}}_{k|k-1}=S_{y_{k|k-1}}S_{y_{k|k-1}}^{T}{\hat{x}}_{k|k-1},\\ \;Y_{k|k-1}=S_{y_{k|k-1}}S_{y_{k|k-1}}^{T},\\ \;\left\{\begin{array}{c} S_{y,k}^{0}=S_{y_{k|k-1}}\\ {\hat{x}}_{k}^{0}={\hat{x}}_{k|k-1} \end{array}\right., \end{array}$
3. UpdateFor the j-th iteration:
(1)Calculate the volume points used to measure renewal:
$\begin{array}{c} \;S_{k|k-1}^{\left(j\right)}=S_{y_{k|k-1}}^{(j)-T},\\ \;X_{i,k\left|k-1\right.}^{\left(j\right)}={\hat{x}}_{k\left|k-1\right.}^{\left(j\right)}+S_{k\left|k-1\right.}^{\left(j\right)}\xi_{i},\\ \;{\tilde{\chi }}_{k|k-1}^{\left(j\right)}=\sqrt{w_{i}}\left[{\hat{X}}_{1,k\left|k-1\right.}^{\left(j\right)}-{\hat{x}}_{k|k-1}^{\left(j\right)},{\hat{X}}_{2,k\left|k-1\right.}^{\left(j\right)}-{\hat{x}}_{k|k-1}^{\left(j\right)},\cdots ,{\hat{X}}_{2n,k\left|k-1\right.}^{\left(j\right)}-{\hat{x}}_{k|k-1}^{\left(j\right)}\right]. \end{array}$

(2)$\mathrm{Calculate}\;\mathrm{the}\;\mathrm{predicted}\;\mathrm{value}\;\mathrm{of}\;\mathrm{the}\;\mathrm{measurement}\;{\;\hat{z}}_{k|k-1}$
$\begin{array}{c} \;Z_{i,k}^{\left(j\right)}=h\left(X_{i,k}^{\left(j\right)}\right),\\ \;{\hat{z}}_{k}^{\left(j\right)}=\sum w_{i}Z_{i,k}^{\left(j\right)}.\\ \;Z_{k}^{\left(i\right)}=\sqrt{w_{i}}\left[Z_{1,k}^{\left(j\right)}-{\hat{z}}_{k}^{\left(j\right)},Z_{2,k}^{\left(j\right)}-{\hat{z}}_{k}^{\left(j\right)},\cdots ,Z_{2n,k}^{\left(j\right)}-{\hat{z}}_{k}^{\left(j\right)}\right]. \end{array}$

(3)Construct a linear regression model:
$\begin{array}{c} \;G_{a,k}^{\left(j\right)}={\tilde{\chi }}_{k}^{\left(j\right)}Z_{k}^{\left(i\right)}Y_{k}^{\left(j\right)},\\ \;G_{b,k}^{\left(j\right)}={\hat{z}}_{k}^{\left(j\right)}-G_{a,k}{\hat{x}}_{k}^{\left(j\right)},\\ \;S_{R_{k|k-1}^{*}}^{\left(j\right)}=Z_{k|k-1}^{\left(j\right)}-G_{a,k}^{\left(j\right)}S_{k|k-1}^{\left(j\right)}+S_{R_{k},}\\ \;\gamma_{k}^{\left(j\right)}=\left[\begin{array}{cc} S_{y_{k|k-1}}^{\left(j\right)} & 0\\ 0 & S_{R_{k|k-1}^{*}}^{\left(j\right)} \end{array}\right],\\ \;\beta_{k}^{\left(j\right)}=\gamma_{k}^{\left(j\right)}\left[\begin{array}{c} {\hat{x}}_{k}^{\left(j\right)}\\ z_{k}-G_{b,k}^{\left(j\right)} \end{array}\right],\\ \;H_{k}^{\left(j\right)}=\gamma_{k}^{\left(j\right)}\left[\begin{array}{c} E\\ G_{a,k}^{\left(j\right)} \end{array}\right],\\ \;{\tilde{\xi }}_{k}^{\left(j\right)}=\beta_{k}^{\left(j\right)}-H_{k}^{\left(j\right)}{\hat{x}}_{k|k-1}^{\left(j\right)},\\ \;\left[\begin{array}{cc} {\bar{\Lambda }}_{x,k}^{\left(j\right)} & 0\\ 0 & {\bar{\Lambda }}_{y,k}^{\left(j\right)} \end{array}\right]=\psi \left({\tilde{\xi }}_{k}^{\left(j\right)}(i)\right). \end{array}$

(4)Construct a robust information filtering framework:
$\begin{array}{c} \;\left\{\begin{array}{c} S_{m,y_{k|k-1}}^{\left(j\right)}={\left({\bar{\Lambda }}_{x,k}^{\left(j\right)}\right)}^{\frac{1}{2}}S_{y_{k|k-1}}^{\left(j\right)}\\ S_{m,R_{k|k-1}^{*}}^{\left(j\right)}={\left({\bar{\Lambda }}_{y,k}^{\left(j\right)}\right)}^{\frac{1}{2}}S_{R_{k|k-1}^{*}}^{\left(j\right)} \end{array}\right.,\\ \;\left\{\begin{array}{c} \left[S_{m,y_{k|k-1}}^{\left(j\right)},G_{a,k}^{\left(j\right)},S_{m,R_{k|k-1}^{*}}^{\left(j\right)}\right]{\overset{SVD}{\to }U}_{Y_{k}^{\left(j\right)}}D_{Y_{k}^{\left(j\right)}}{\left(V_{Y_{k}^{\left(j\right)}}\right)}^{T}\\ S_{y_{k}}^{\left(j\right)}\overset{SVD}{\to }U_{Y_{k}^{\left(j\right)}}\sqrt{D_{Y_{k}^{\left(j\right)}}} \end{array}\right.,\\ \;S_{k}^{\left(j\right)}=S_{y_{k}}^{(j)-T},\\ \;,\;i_{k}^{\left(j\right)}={\left(G_{a,k}^{\left(j\right)}\right)}^{T}S_{m,R_{k|k-1}^{*}}^{\left(j\right)}{\left(S_{m,R_{k|k-1}^{*}}^{\left(j\right)}\right)}^{T}\left(z_{k}-G_{b,k}\right),\\ \;{\hat{y}}_{k}^{\left(j+1\right)}={\hat{y}}_{k\left|k-1\right.}^{\left(j\right)}+i_{k}^{\left(j\right)},\\ \;\left\{\begin{array}{c} {\hat{P}}_{m,k}^{\left(j+1\right)}={\left(S_{y,k}^{\left(j\right)}S_{y,k}^{(j)T}\right)}^{-1}\\ {\hat{x}}_{k}^{\left(j+1\right)}={\hat{P}}_{m,k}^{\left(j+1\right)}{\hat{y}}_{k}^{\left(j+1\right)} \end{array}\right.. \end{array}$

end

### 5.2. Improved Robust Kernel Function

**Figure 2:**
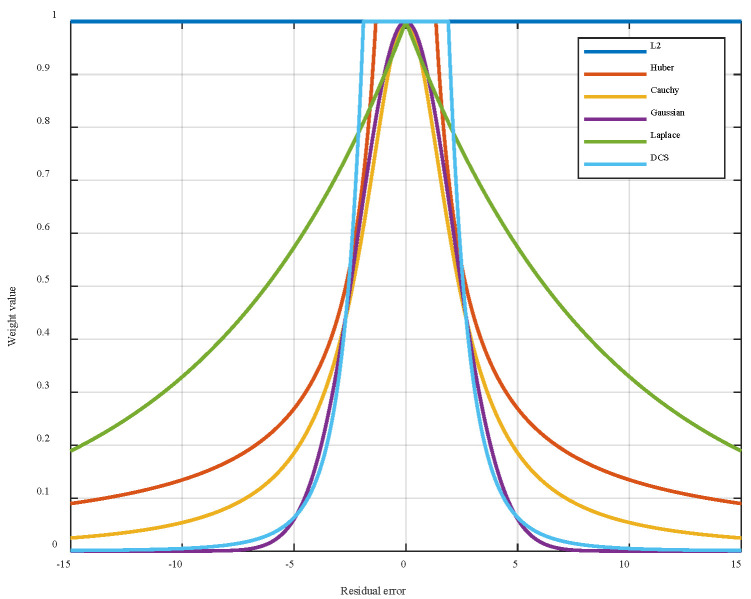
Comparison of different versions of robust kernel weight function.

We adopt the DCS kernel function to replace the conventional Huber kernel. This further mitigates outlier influence. The literature shows that DCS has a theoretical relationship with IRLS through robust M-estimation [34]. When integrated into M-estimation as a kernel function, DCS can be mathematically expressed by Equation (11). At present, in the range of outlier values, the performance of each robust kernel function differs [39,40,41,42,43]. In particular, it can be observed from the weight kernel functions of various versions in Figure 2 that only the Huber and DCS kernel functions still complete state estimation based on L2- norm criteria, which preserves the optimality of the L2- norm in the Gaussian noise environment.

Figure 2 presents a comparative plot of weight functions from widely adopted robust kernel functions. As observed, the DCS and Gaussian kernel functions demonstrate pronounced suppression effects on large residual errors, substantiating their robust outlier rejection capabilities.

When there is a large residual, the Huber weight function has a heavy tail, which indicates that the Huber function cannot suppress the influence of data in the outlier range on the estimator. The DCS function has only one adjustment parameter, and the influence function decreases rapidly and almost approaches 0 in the outlier range. This indicates that the DCS kernel function can eliminate outlier values and the influence of outlier values on the estimation results, further strengthening the robustness of the estimator. To eliminate the influence of strong non-Gaussian noise while preserving the estimation accuracy of the estimator in Gaussian noise, DCS is selected as the robust kernel function of the proposed algorithm framework.

From Equations (37) and (38), it can be observed that the MMCKF algorithm can be interpreted as a superposition of robust kernel functions from the perspective of robust regression. However, directly combining the DCS kernel with the maximum correntropy cost function would compromise its L2- norm compliance in small residual regimes, potentially reducing estimation accuracy under Gaussian conditions. Similar limitations apply to other L2-preserving IMMCIF variants. Modified weight functions addressing this issue, including MCC-Huber and MCC-DCS, are detailed in Table 1.

**Figure 3:**
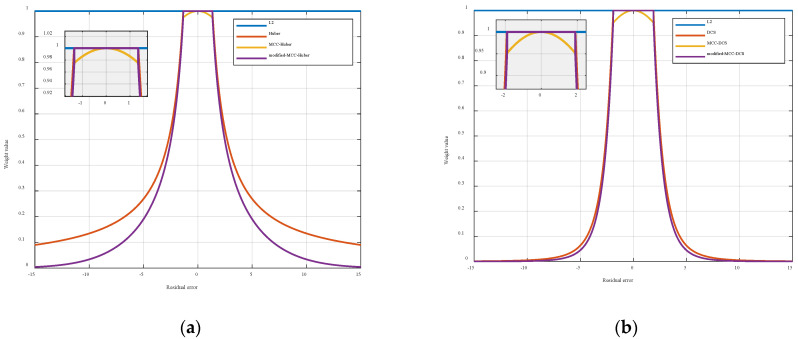
Comparison of different versions of Huber and DCS kernel weight functions. (**a**) Comparison of different versions of Huber kernel weight function. (**b**) Comparison of different versions of DCS kernel weight function. The analysis reveals that maximum correntropy-based kernels achieve enhanced outlier rejection while maintaining L2- norm compliance in small residual ranges. This dual capability enables better adaptability to noise intensity variations and improved estimation accuracy compared to conventional implementations.

Figure 3 presents a comparative analysis of weight characteristics among three kernel functions: the original kernel, the maximum correntropy-based kernel, and its enhanced version. The comparison demonstrates that, while the Huber kernel with maximum correntropy cost function shows notable improvement in outlier rejection capability (evident from its heavier-tailed distribution), the DCS kernel exhibits inherently stronger outlier resistance with less significant enhancement through correntropy modification.

Notably, the improved methodology preserves the L2- norm criterion of the original kernel function, ensuring estimation accuracy in Gaussian environments. Within outlier ranges, the enhanced estimator demonstrates accelerated attenuation (approaching zero asymptotically), outperforming the original kernel in outlier rejection efficiency.

## 6. Simulation and Analysis

The seeker system noise in the strapdown guidance system was set as the Gaussian white noise based on the ideal model. Non-Gaussian radar observations are typically encountered because of the interference between reflections on the target surface. In the seeker imaging guidance system, the measurement noise was considered non-Gaussian white noise in this simulation. The probability distribution is expressed using the following equation:

(45) $$f(\omega )=\frac{1-\varepsilon }{\sqrt{2\pi }\sigma_{1}}exp\left[-\frac{{\left(\omega /\sigma_{1}\right)}^{2}}{2}\right]+\frac{\varepsilon }{\sqrt{2\pi }\sigma_{2}}exp\left[-\frac{{\left(\omega /\sigma_{2}\right)}^{2}}{2}\right],$$

where ε denotes the contamination rate, and $\sigma_{1}$ and $\sigma_{2}$ denote the standard deviations of the measurement noise and contaminated noise, respectively.

**Figure 4:**
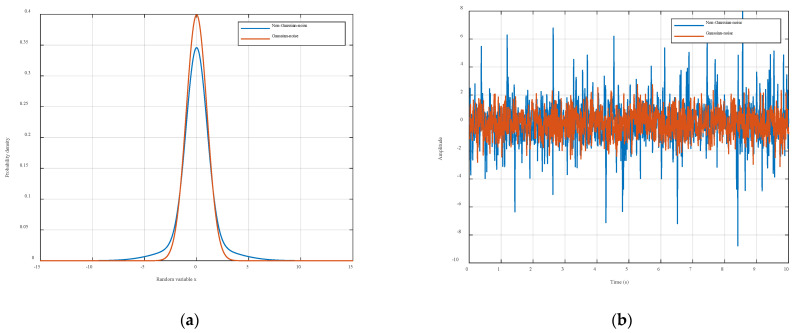
Comparison between standard Gaussian white noise and non-Gaussian noise. (**a**) Gaussian and non-Gaussian densities for a scalar case. (**b**) Comparison of standard Gaussian white noise and non-Gaussian noise random numbers.

A comparison between standard Gaussian white noise and non-Gaussian noise is illustrated in Figure 4. Standard Gaussian white noise has symmetric amplitude distribution. It shows rapid tail decay. Extreme values are rare. Its amplitude is statistically independent over time and lacks impulsive features. In contrast, non-Gaussian noise typically exhibits a heavy-tailed distribution, where the probability density function (PDF) decays more slowly than the Gaussian case, leading to significantly higher probabilities of extreme values. Additionally, non-Gaussian noise demonstrates impulsive behavior, manifested as transient large-amplitude spikes [44,45].

Figure 4 presents the probability density functions (PDFs) and random number samples of standard Gaussian white noise and non-Gaussian noise. The comparative PDF analysis reveals that standard Gaussian white noise exhibits rapid tail decay with negligible outlier probabilities, whereas non-Gaussian noise shows pronounced heavy-tailed characteristics. Furthermore, the random number samples highlight the absence of impulsive behavior in standard Gaussian white noise, in sharp contrast to the non-Gaussian counterpart, which displays distinct pulsed patterns characterized by short-duration, high-amplitude spikes.

At this juncture, the simulation experiment was conducted under identical initial conditions to compare the experimental results of the conventional SRIF, IMCSRIF (high estimation accuracy for non-Gaussian noise), and IMMCSRIF. To facilitate a visual assessment of the accuracy of the filter, RMSE was employed. The RMSE, a commonly used statistical metric, quantifies the disparity between the predicted values and actual observations. It computes the average difference between the predicted values and actual observations and subsequently converts them into the same units as the original observed data. The RMSE for each filter at each time instant is calculated as follows:

(46) $$RMSE_{k,i}=\sqrt{\frac{1}{MC}\sum_{m=1}^{MC}{\left({\hat{x}}_{k,i}^{m}-x_{k,i}^{m}\right)}^{2}},$$

where k denotes the k-th instant, i denotes the i-th component of the state vector, and MC denotes the number of Monte Carlo simulations. ARMSE for each filter is defined as follows, which represents the average of ${\mathrm{RMSE}}_{i}$ from instant $k_{0}$ to instant $k_{n}$:

(47) $${\mathrm{ARMSE}}_{i}=\frac{1}{k_{n}-k_{0}}\sum_{k=k_{0}}^{k_{n}}{\mathrm{RMSE}}_{k,i},$$

To quantify the assessment of filtering efficiency, a metric termed the filtering relative efficiency ($E_{i}$) is defined as follows:

(48) $$E_{i}=\frac{{ARMSE}_{best}}{{ARMSE}_{i}},$$

where

- ${ARMSE}_{best}$ denotes ARMSE achieved by the filtering algorithm exhibiting the highest estimation accuracy.
- ${ARMSE}_{i}$ denotes the ARMSE of the specific filtering algorithm under evaluation.

The interpretation of this metric is as follows:

As $E_{i}$ → 100%, the performance of the evaluated algorithm asymptotically approaches that of the benchmark algorithm, indicating comparable efficiency. As $E_{i}$ → 0, the evaluated algorithm demonstrates significantly lower efficiency relative to the benchmark algorithm.

Through 300 Monte Carlo shooting simulations, the following results are obtained. Table 2 and Table 3 present the parameters of the initial conditions. ARMSE results corresponding to RMSE under Gaussian and non-Gaussian noise for different algorithm frameworks are shown in Table 4.

### 6.1. Comparative Study Under Gaussian Observation Noise

**Figure 5:**
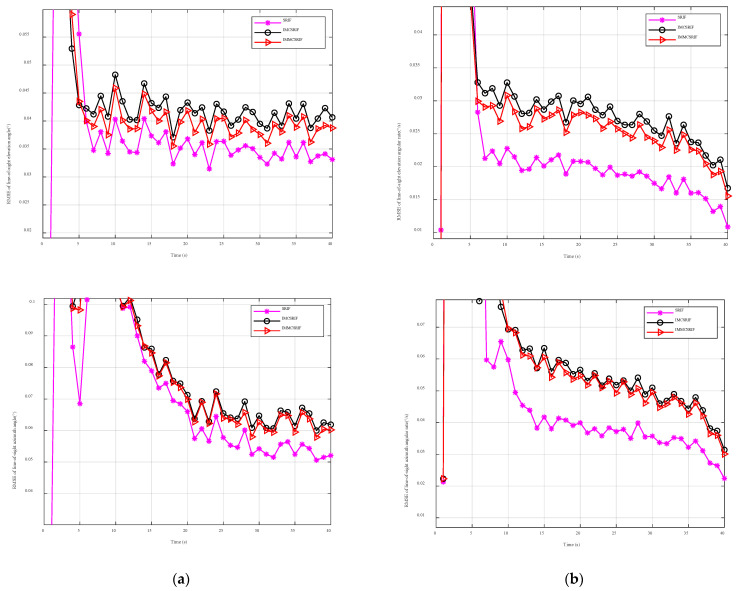
Dependence for RMSE elevation angle and angular rate results in the case of Gaussian noise. (**a**) RMSE elevation angle in the case of Gaussian noise. (**b**) RMSE elevation angle rate in the case of Gaussian noise.

To evaluate the impact of the improved robust kernel function on estimation accuracy, Monte Carlo simulations were performed under Gaussian observation noise. The filtering algorithm was set to three iterations. In the comparative experiments, SRIF is a Gaussian filter based on the assumption of normally distributed noise. Other filters (IMMCSRIF, IMCSRIF) adopt robust filtering principles. Figure 5 shows the statistical RMSE results for line-of-sight angles and angular rates. SRIF demonstrates higher estimation accuracy than IMMCSRIF and IMCSRIF. This is because replacing the L2- norm criterion with a kernel function reduces filtering efficiency in Gaussian noise environments. However, by adjusting the kernel width parameter, the robust filter can achieve 95% relative efficiency compared to the standard Kalman filter under standard normal noise. Key conclusion: Robust filtering efficiency decreases under Gaussian noise.

Figure 5 presents comparative filtering accuracy plots of line-of-sight angles and angular rates under Gaussian noise conditions. The analytical results demonstrate that the SRIF employing L2- norm criterion achieves superior estimation accuracy, as kernel function approaches replacing the L2- criterion incur accuracy degradation in Gaussian environments. Notably, the IMMCSRIF maintains L2- compliance within small residual ranges, thereby attaining enhanced precision compared to the IMCSRIF.

A comparison between IMCSRIF and IMMCSRIF reveals that IMMCSRIF achieves higher estimation accuracy in Gaussian noise. This improvement stems from its Huber kernel function, which enforces the L2- norm criterion under small residuals. To evaluate the filtering accuracy quantitatively, we choose the relative ratio of the ARMSE results as the evaluation criterion. This can be calculated from the data of the filtering algorithm ARMSE in Table 4 and the definition of the filtering relative efficiency that the relative filtering efficiency of IMCSRIF at the line-of-sight angle and line-of-sight angle rate is 84.33%, 88.56%, 68.55%, and 70.72%, respectively. The corresponding values of the IMMCSRIF were 88.74%, 90.23%, 73.28%, and 72.98%, respectively.

### 6.2. Comparative Study Under Non-Gaussian Observation Noise

**Figure 6:**
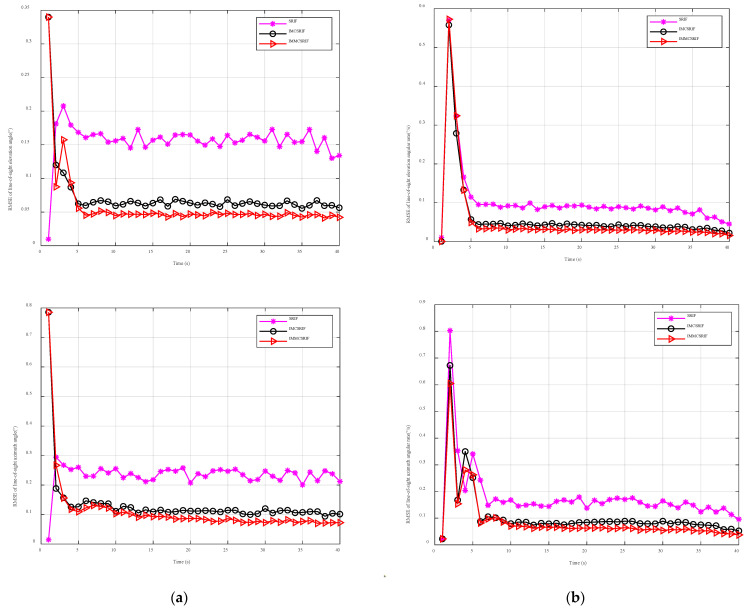
Dependence for RMSE elevation angle and angular rate results in the case of η=10 (**a**). (**b**) RMSE elevation angle rate in the case of η=10.

To validate the effectiveness of our proposed algorithm, we compare the filtering performance of SRIF, IMCSRIF, and IMMCSRIF under non-Gaussian observation noise. Figure 6 and Table 4 summarize the RMSE and ARMSE results for line-of-sight angles and angular rates. Here, *η* represents the standard deviation of contaminated measurement noise, reflecting the contamination intensity. When η=10, the noise exhibits significant non-Gaussianity due to its large standard deviation.

Figure 6 displays comparative filtering accuracy plots of line-of-sight angles and angular rates under severe non-Gaussian noise conditions. The results indicate three key observations: (1) The L2- norm-based SRIF exhibits the poorest estimation accuracy due to outlier susceptibility, (2) robust kernel-based filters demonstrate effective outlier suppression through adaptive weighting mechanisms, and (3) most significantly, the IMMCSRIF achieves superior precision by implementing rapidly decaying weight tails that approach zero, thereby significantly enhancing outlier rejection efficacy compared to the IMCSRIF framework.

As shown in Figure 6, SRIF’s accuracy deteriorates rapidly because it relies on a Gaussian distribution assumption. In contrast, IMCSRIF and IMMCSRIF—both employing robust strategies—effectively suppress non-Gaussian noise. Notably, IMMCSRIF achieves the highest accuracy in both angle and angular rate estimation.

Next, a quantitative analysis is conducted, and IMMCSRCIF is selected as a reference. The specific conclusions are as follows. Using IMMCSRCIF as the baseline filter, the filtering relative efficiency of SRIF compared to this benchmark is 29.89%, 34.69%, 35.17%, and 38.63% for line-of-sight angle and line-of-sight angle rate, respectively. In contrast, the relative efficiency of IMCSRCIF is 75.21%, 73.41%, 75.88%, and 75.17% for the same parameters.

The reason is that the estimation accuracy of the seeker with non-Gaussian noise cannot be accurately estimated using SRIF assuming a normal noise distribution, but the estimation accuracy can be improved by using a robust regression strategy. However, IMCSRIF cannot select the best kernel width based on the working environment of the seeker, which leads to a reduction in filtering accuracy. The difference is that IMMCSRIF introduces the L2- norm criterion when handling small residuals, and its estimation accuracy is better than that of IMCSRIF using the maximum correlation entropy estimation method. Simultaneously, when handling large residuals, the combination of generalized M estimation theory and maximum correlation entropy shows a stronger inhibition of outlier values, and the decline is faster and almost approaches 0 in the range of outlier values, indicating that the method has a strong ability to eliminate outlier values, and further strengthens the robustness of the estimator.

### 6.3. Kernel Comparison

**Figure 7:**
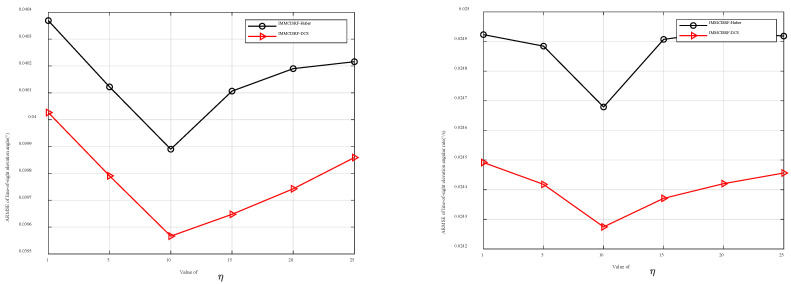

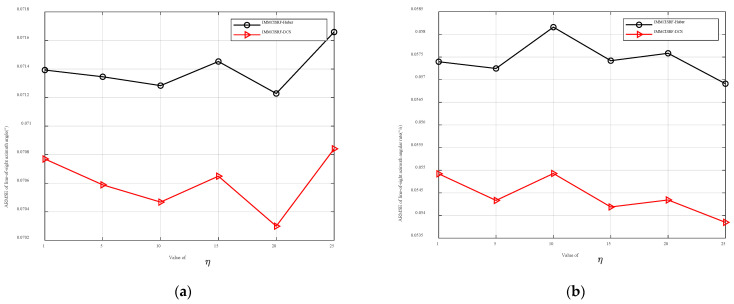
Dependence for ARMSE elevation angle and angular rate results in different η cases. (**a**) ARMSE elevation angle for different η values. (**b**) ARMSE elevation angle rate for different η values.

**Figure 8:**
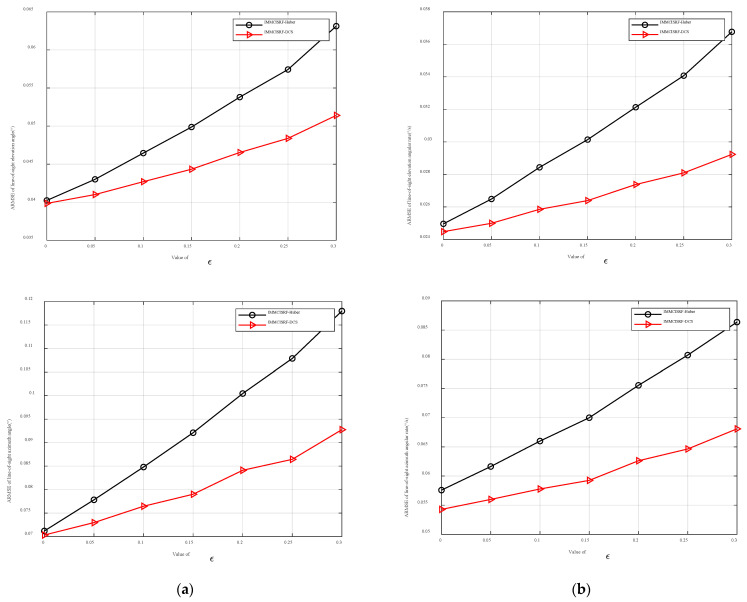
Dependence for ARMSE elevation angle and angular rate results in different ε cases. (**a**) ARMSE elevation angle for different ε values. (**b**) ARMSE elevation angle rate for different ε values.

To explore the influence of robust kernel function selection on seeker estimation accuracy, the Huber kernel function and DCS kernel function with the L2- norm criterion are selected for comparison. The robustness of the kernel function in different environments is observed by setting different pollution measurement noise covariances and pollution degrees. Figure 7 depicts the accuracy errors of the line-of-sight angle and line-of-sight angle rate for different filtering methods under varying pollution probability ε=0.2 and covariance of pollution measurement noise η=1, 5, 15, 20, and 25. Similarly, Figure 8 illustrates the accuracy errors of the line-of-sight angle and line-of-sight angle rate for different filtering methods under different covariances of pollution measurement noise η=10 and pollution probability ε = 0, 0.05, 0.1, 0.15, 0.2, 0.25, and 0.3.

Figure 7 presents comparative estimation accuracy plots of line-of-sight angles and angular rates across varying noise intensities. The analysis reveals three principal findings: (1) The IMMCISRF framework’s Huber and DCS robust filters maintain high-precision estimation under all noise conditions, demonstrating exceptional environmental adaptability; (2) This superior performance stems from their dual-mode operation: L2- norm compliance in small residual ranges coupled with rapid weight decay approaching zero for large residuals, ensuring simultaneous outlier rejection and optimal noise adaptation; (3) Comparative evaluation shows the DCS variant achieves precision superiority through extended L2- compliance domains and accelerated weight function attenuation beyond critical residual thresholds.

Through the ARMSE comparison experiment, it can be observed that, because the comparison algorithm adopts the IMMCSRIF algorithm framework, IMMCSRIF using the Huber kernel function, and IMMCSRIF using the DCS kernel function have high estimation accuracy under various noise covariances and pollution probability environments. Both algorithms meet the requirement of seeker estimation accuracy when the noise statistics are unstable and the statistical information is unknown. IMMCSRIF-DCS outperforms IMMCSRIF-Huber. The DCS kernel achieves higher accuracy across noise covariances and contamination probabilities.

This is because, although both Huber kernel functions and DCS kernel functions have L2- norm criteria, DCS kernel functions have a wider range of L2- norm criteria than Huber kernel functions over a small residual range. Its characteristics ensure that the estimation accuracy is better than that of the Huber kernel function in the case of Gaussian noise. In the range of large residuals, the DCS kernel function decreases faster than the Huber kernel function, indicating that the DCS kernel function can eliminate outlier values.

Figure 8 presents a comparative analysis of estimation accuracy for line-of-sight angle and angular rate under varying noise contamination probabilities. As demonstrated, the Huber and DCS robust filtering methods implemented through the IMMCISRF framework maintain superior estimation precision across different noise contamination levels, exhibiting robust adaptability. This phenomenon shares the same underlying mechanism as illustrated in Figure 7. Notably, distinct from Figure 7, the proposed algorithm manifests gradual degradation in estimation accuracy with increasing noise contamination probabilities. This performance deterioration stems from the fundamental principle of robust kernel functions that strategically attenuate the weighting of large-residual samples to mitigate outlier impacts. However, when anomalous measurements cease to be sparse, the weight attenuation mechanism inevitably suppresses excessive normal samples through misclassification, consequently permitting contamination data to adversely influence estimation accuracy.

In summary, although the two comparison algorithms adopt the IMMCSRIF algorithm framework, both show high estimation accuracy under various noise covariances and pollution probability environments. However, the DCS kernel function can better adapt to noise instability and the uncertainty of noise statistics in the seeker operating environment than the Huber kernel function because the DCS kernel function guarantees a larger range of the L2- norm criteria in the small residual and the weight function decreases faster in the large residual range. This shows higher precision and stronger robustness in the estimation.

## 7. Discussion

To address non-Gaussian noise with unknown statistics in strapdown seeker measurements, this study integrates generalized M-estimation with IMCSRIF. SVD is incorporated to mitigate estimation divergence risks caused by improper kernel width selection. In all cases of non-Gaussian noise, the robust filtering performance of the IMMCSRIF filter framework is significantly improved. Simulation results demonstrate that the proposed framework achieves significantly higher estimation accuracy than IMCSRIF under both Gaussian and non-Gaussian noise conditions, as evidenced by RMSE comparisons. The proposed algorithm framework presents higher estimation accuracy and stronger robustness. To enhance adaptability to dynamic and uncertain strapdown seeker noise conditions, IMMCSRIF is reformulated through robust regression principles as a robust filtering architecture with composite kernel functions; concurrently, a novel kernel optimization framework is developed to strengthen noise immunity. IMMCSRIF is reformulated via robust regression. Composite kernel functions enhance adaptability. An optimized kernel design further strengthens robustness.

This method maintains the L2- norm criterion while enhancing the decay rate of the robust kernel weight function for large residuals. Simulation results demonstrate that the proposed filtering framework achieves superior accuracy in both Gaussian and non-Gaussian environments. This study employs the Huber kernel function and DCS kernel function as case studies to analyze how robust kernel selection impacts estimation precision. Comparative simulations reveal that the IMMCSRIF-DCS algorithm outperforms IMMCSRIF-Huber in estimation accuracy, benefiting from its extended L2- norm criterion range and accelerated decay characteristics for large residuals. This indicates that the adoption of the IMMCSRIF-DCS algorithm enables the strapdown seeker to achieve high state estimation accuracy in both Gaussian and non-Gaussian environments, demonstrating robust adaptability to variable working conditions. Due to the limited knowledge reserve and research time, the proposed method has the possibility of further improvement. The algorithm framework proposed in this paper is still unable to fully adapt to the changeable working environment of the strapdown seeker. There are many adaptive filtering methods at present. How to use the adaptive filtering method to rewrite the proposed algorithm framework to adapt to the changeable working environment of the strapdown seeker is the direction of future research.

## Figures and Tables

**Table 1:** Different versions of Huber and DCS kernel functions.

Name	Kernel Function	MCC-Kernel Function	Improved MCC-Kernel Function
Huber	$\psi_{H}(\xi_{k,i})=\left\{\begin{array}{c} \;1,\;for\xi_{k,i}<\mu \\ \frac{\mu }{\left|\xi_{k,i}\right|},\;for\xi_{k,i}\geq \mu \end{array}\right.$,	$\psi_{MCC-H}(\xi_{k,i})=\left\{\begin{array}{c} G_{\sigma }\left(\xi_{k,i}\right),\;for\;\xi_{k,i}<\mu \\ \frac{\mu G_{\sigma }\left(\xi_{k,i}\right)}{\left|\xi_{k,i}\right|},\;for{\;\xi }_{k,i}\geq \mu \end{array}\right.$ ,	$\psi_{MCC-H}^{\left(mod\right)}(\xi_{k,i})=\left\{\begin{array}{c} \;1,\;for{\;\xi }_{k,i}<\mu \\ \frac{\mu G_{\sigma }\left(\xi_{k,i}\right)}{\left|\xi_{k,i}\right|},\;for\;\xi_{k,i}\geq \mu \end{array}\right.$ ,
DCS	$\psi_{D}\left(\xi_{k,i}\right)=\left\{\begin{array}{c} \;1,for\xi_{k,i}^{2}<\theta \\ \frac{4\theta }{{\left(\theta +\xi_{k,i}^{2}\right)}^{2}},for\xi_{k,i}^{2}\geq \theta \end{array}\right.,$	$\psi_{MCC-D}\left(\xi_{k,i}\right)=\left\{\begin{array}{c} \;G_{\sigma }\left(\xi_{k,i}\right),for{\;\xi }_{k,i}^{2}<\theta \\ \frac{4\theta G_{\sigma }\left(\xi_{k,i}\right)}{{\left(\theta +\xi_{k,i}^{2}\right)}^{2}},for\;\xi_{k,i}^{2}\geq \theta \end{array}\right.,$	$\psi_{MCC-D}^{\left(mod\right)}(\xi_{k,i})=\left\{\begin{array}{c} \;1,\;for\;\xi_{k,i}^{2}<\theta \\ \frac{4\theta G_{\sigma }\left(\xi_{k,i}\right)}{{\left(\theta +\xi_{k,i}^{2}\right)}^{2}},\;for\;\xi_{k,i}^{2}\geq \theta \end{array}\right.$ ,

**Table 2:** Missile and target initial configuration conditions.

Parameter	Corresponding Value
Initial missile position	(0 km, 0 km, 0 km)
Initial target position	(10 km, 5 km, 10 km)
Initial missile velocity	(0.6 km/s, 0, 0)
Initial target velocity	(0.364 km/s, 0, 0.21 km/s)
Discrete sampling period	100 ms

**Table 3:** Initial settings for filters.

Parameter	Corresponding Value
Initial covariance matrix of filters	$P_{0}=diag[2.7\times 10^{-8},2\times 10^{-6},2\times 10^{-4},4.5\times 10^{-3}]$
Covariance matrix of process noise	$Q_{k}=diag\left(\left[4\times 10^{-6},4\times 10^{-5},4\times 10^{-6},4\times 10^{-5}\right]\right)$
Covariance matrix of observation noise	$R_{k}=diag\left(\left[2.2\times 10^{-6},2.2\times 10^{-6}\right]\right)$
Initial state vector	$x_{0}=[0.34;1\times 10^{-3};-0.8;1\times 10^{-3}]$
Perturbing parameter in the case of non-Gaussian noise	0.2

**Table 4:** ARMSE results of line-of-sight angle and line-of-sight angular velocity for different algorithms.

Algorithms	Gaussian Noise				Non-Gaussian Noise (*η*)			
	$q_{\gamma }$	${\dot{q}}_{\gamma }$	$q_{\lambda }$	${\dot{q}}_{\lambda }$	$q_{\gamma }$	${\dot{q}}_{\gamma }$	$q_{\lambda }$	${\dot{q}}_{\lambda }$
SRIF	**0.0339**	**0.0170**	**0.0619**	**0.0343**	0.1522	0.0764	0.2334	0.1434
IMCSRIF	0.0402	0.0248	0.0699	0.0485	0.0605	0.0361	0.1082	0.0737
IMMCSRIF	0.0382	0.0232	0.0686	0.0470	**0.0455**	**0.0265**	**0.0821**	**0.0554**

## Data Availability

No new data were created or analyzed in this study. Data sharing is not applicable to this article.

## Abbreviations

The following abbreviations were used in this manuscript:

ARMSE	Average root mean squared error
DCS	Dynamic covariance scaling
EKF	Extended Kalman filter
GML	Generalized maximum likelihood estimation
IMCIF	Iterative maximum correlation entropy information filter
IMMCSRIF	Iterative maximum correlation entropy square root information filter based on a generalized M estimation
IRLS	Iteratively reweighted least squares
ITL	Information theory learning
MCC	Maximum correlation entropy criteria
MCC-DCS	Maximum correntropy criterion with dynamic covariance scaling
MCC-Huber	Maximum correntropy criterion with Huber
MEE	Minimum error entropy
MMCKF	Maximum correlation entropy Kalman filter based on a generalized M estimation
MMCSRIF	Maximum correlation entropy square root information filter based on a generalized M estimation
RMSE	Root mean square error
SRIF	Square root information filter
SVD	Singular value decomposition
UKF	Unscented Kalman filter
